# How the ecology and evolution of the COVID‐19 pandemic changed learning

**DOI:** 10.1002/ece3.6937

**Published:** 2020-10-29

**Authors:** Marcus A. Lashley, Miguel Acevedo, Sehoya Cotner, Christopher J. Lortie

**Affiliations:** ^1^ Department of Wildlife Ecology and Conservation University of Florida Gainesville FL USA; ^2^ Department of Biology Teaching and Learning University of Minnesota‐Twin Cities Minneapolis MN USA; ^3^ Department of Biology York University Toronto ON Canada

## Abstract

The coronavirus disease 2019 (COVID‐19) pandemic introduced an abrupt change in human behavior globally. Here, we discuss unique insights the pandemic has provided into the eco‐evolutionary role of pathogens in ecosystems and present data that indicates the pandemic may have fundamentally changed our learning choices. COVID‐19 has indirectly affected many organisms and processes by changing the behavior of humans to avoid being infected. The pandemic also changed our learning behavior by affecting the relative importance of information and forcing teaching and learning into a framework that accommodates human behavioral measures to avoid disease transmission. Not only are these indirect effects on the environment occurring through a unique mechanistic pathway in ecology, the pandemic along with its effects on us provides a profound example of the role risk can play in the transmission of information between the at risk. Ultimately, these changes in our learning behavior led to this special issue “Taking learning online in Ecology and Evolution.” The special issue was a call to the community to take learning in new directions, including online and distributed experiences. The topics examined include a significant component of DIY ecology and evolution that is experiential but done individually, opportunities to use online tools and apps to be more inclusive, student‐focused strategies for teaching online, how to reinvent conferences, strategies to retain experiential learning safely, emerging forms of teaching such as citizen science, apps and podcasting, and ideas on how to accommodate ever changing constraints in the college classroom, to name a few. The collective consensus in our fields is that these times are challenging but we can continue to improve and innovate on existing developments, and more broadly and importantly, this situation may provide an opportunity to reset some of the existing practices that fail to promote an effective and inclusive learning environment.

## Graphical abstract

COVID‐19 has indirectly affected many species and processes by causing an abrupt shift in human behavior globally. Ultimately, these shifts in behavior have affected how we value information and how we teach one another.

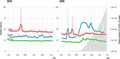

## 1 INTRODUCTION

Theory in ecology and evolution helps us understand patterns and processes in natural systems and in human society. In this manuscript, we discuss consequences the coronavirus disease 2019 (COVID‐19) pandemic has generated that can provide unique insights into the role pathogens and fear can play in ecosystems and how using humans as a model provides us a unique opportunity to understand those mechanisms. Herein, we discuss that by changing our behavior, the pandemic has generated indirect effects through a unique pathway in ecology and evolution, and we present novel data to show that a key way that the pandemic has changed our behavior is to alter how we value and seek information, and also how we transmit information to one another. Our hope was to highlight these seemingly disparate aspects of the pandemic to bring attention to their ultimate role in affecting how and what we learn and the changes it fosters in society.

## 2 A UNIQUE PATHWAY FOR INDIRECT EFFECTS OF COVID‐19

The COVID‐19 pandemic is a profound example of a pathogen generating indirect effects by altering host behavior. Interestingly, this may be the only example of those indirect effects generated by a pathogen being primarily related to behavioral changes of individuals of the host species who were not infected. That is, in the current literature base indirect effects have only been linked to changes in behavior of infected individuals. Six months after the first documented infection, the pathogen had infected only a small proportion of the global population (15 million confirmed cases as of 30 July 2020; Dong et al., 2020), yet it had resulted in widespread changes in the way billions of people live (e.g., Corlett et al., 2020; Gössling et al., 2020; Rutz et al., 2020). These behavioral changes, arising primarily to avoid infection, have had cascading indirect effects of the pathogen on the environment such as decreased concentrations of some greenhouse gasses in the atmosphere, changes in air quality and environmental pollution, and decreases in anthropogenic sound (e.g., Chen et al., 2020; Corlett et al., 2020; Isaifan, 2020; Zambrano‐Monserrate et al., 2020). Plus, the decrease in human activity (i.e., anthropause) has provided a strong natural experimental framework for researchers to uncover how humans affect wildlife communities (Rutz et al., 2020). Based on ecological and evolutionary theory, these indirect effects from pathogens changing the behavior of their hosts should be prevalent in nature because we know organisms—humans and many other species—commonly change their behavior in response to pathogens—either as a result of getting infected, or to avoid getting infected (Curtis, 2014; Weinstein et al., 2018). However, a recent and comprehensive review (i.e., Buck & Ripple, 2017) only uncovered one example where a pathogen generated indirect effects by infecting the host (i.e., a pathogen indirectly affected prey abundance by reducing the feeding rate in the predatory crawfish it infected; Haddaway et al., 2012) and found no published examples of indirect effects from a pathogen generated by behavioral avoidance of getting infected (Buck & Ripple, 2017). Indeed, indirect effects stemming from host avoidance of parasites are rare as well (Buck, 2019). Given that the majority of people changing their behavior have not been infected, COVID‐19 has provided a compelling and rare example of indirect effects by a pathogen being caused by behavioral avoidance of infection. Likewise, COVID‐19 provides us with a stark reminder that humans are a part of, and not separate from, the principle eco‐evolutionary processes that shape our world. We as ecologists and evolutionary biologists can use the pandemic to advance our knowledge base, and also use our knowledge base to inform responses to the pandemic.

## 3 THE PANDEMIC HAS CHANGED WHAT WE SEEK TO LEARN

One of the principle ways the pandemic has affected our behavior was to invoke social distancing as a measure to avoid disease transmission. Interestingly, one consequence of that behavioral shift was to increase reliance in online learning. Online learning is a deep and rich field of research, tools, and ideas. The use of the term “online learning” is not universal and can describe different concepts (Moore et al., 2011). It can refer to learning entirely online or more broadly the use of online tools and virtual spaces to engage and support learning (Wallace, 2003). The pandemic has caused many shifts including behavioral responses to interest in online learning, teaching, and information technology. Some of these changes are driven by necessity, including emergency delivery of teaching content online but they also may stem from fear of infection. Further, interest in online learning can shift because of emotional drivers; to explore this idea, we can turn to data on internet searches (i.e., Google Trends). Predicting human behavior using internet searches is a well‐established field of research in consumer studies (Preis et al., 2013) and other disciplines (Nuti et al., 2014), and the evidence suggests that volume of searches provides a useful predictive guide to the near future in behavior (Goel et al., 2010). This tool estimates the relative interest (or trends) of internet users globally by comparing the relative frequencies of search queries between different time periods, allowing us to infer change in human priorities. There are numerous examples of this approach, and current examples have involved using Google Trends data to examine indicators of private consumption (Vosen & Schmidt, 2011), predicted cryptocurrency market values (Kristoufek, 2013), trading behavior in financial markets (Preis et al., 2013), human health and health care (Heerfordt & Heerfordt, 2020; Nuti et al., 2014), and—more pertinent to this discussion—shifts in consumer choices during the pandemic (Schmidt et al., 2020). Specifically, the Google Trends tool provides data and trend analyses by key terms through time. While the overall predictive accuracy of Google Trends has been challenged by periods of unusually high search prevalence (i.e., ephemeral spikes; Butler, 2013), recent developments have improved its predictive accuracy (Kandula & Shaman, 2019). Our primary aim here was to compare the dynamics of relative interests in different types of information during the pandemic globally using Google Trends (Choi & Varian, 2012). Understanding that limitations exist with Google Trends, we saw an opportunity to frame the concepts developed in this special issue within this larger, global conversation and engagement with information to understand how we fit in to the ecology and evolution of the pandemic but also how our learning environment must change in response.

**Figure 1:**
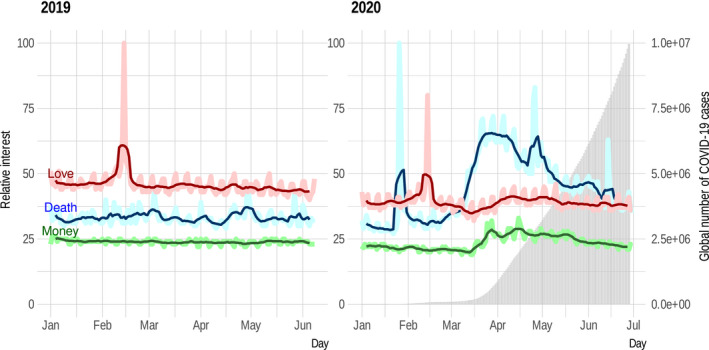
The figure shows how the relative frequency of Google searches on global topics related to money, death, and love changed over time. The panel on the left shows data from 1 January to 30 June 2019 (no COVID‐19), while the panel on the right shows data from 1 January to 30 June 2020 (during the COVID‐19 pandemic). Google Trends does not provide the total number of searchers for a term (Burivalova et al., 2018). They provide an adjusted proportion of searches. The thicker lines correspond to daily values, while the smaller line in the front corresponds to detrended weekly averages. In 2019, Google searches on love were the most common followed by death and then by money. Users' interests remained stable through time except for a spike in searches on love around February 14, which corresponds to Saint Valentine's day. In 2020, the pattern is similar until mid‐March, with the exception of a spike in searches on topics related to death around January 26, which corresponds to increased interest in the death of basketball player Kobe Bryant who died in a helicopter accident. Gray bars represent the global number of confirmed COVID‐19 cases (Dong et al., 2020). Note that as the number of COVID‐19 cases increases, there is a shift in the relative interest in Google searches. There is a small relative increase in searches related to money. Death becomes the top category, switching places with love. Data used in the figure are available at https://github.com/maacevedo/covid19gtrends

To do this, we first examined the relative interests in topics related to risk (“death”), resources (“money”), and reproduction (“love”), three currencies we often value in natural systems in our fields. Google trends indicated dramatic shifts in relative interest between these topics at the onset of the COVID‐19 pandemic. Google searches between the same January–June period in 2019 and then again in 2020 showed that our relative interests in reproduction (Google search global topics related to “love”), risks (Google search global topics related to “death”), and resources (Google search global topics related to “money”) dramatically shifted in 2020 tracking increases in confirmed infections (https://github.com/maacevedo/covid19gtrends). While in 2019, topics of “love” were the most frequently searched of these three, in 2020, with an increasing number of infections, “love” was replaced by topics related to “death” (Figure 1). A shift in relative importance of reproduction and risk of mortality can be expected based on ecological and evolutionary theory on the effects of predation risk (Lima & Dill, 1990) and risk of infection (Weinstein et al., 2018), but we found it interesting that this pattern seems to have emerged in global patterns of online searches in parallel to increasing infections in the pandemic. These changes may stem from the desire to learn about COVID‐19 so that one can better calculate the risk it poses and determine the appropriate behavioral response—especially given that other authors have attributed to the pandemic a heightened level of fear in people (Ahorsu et al., 2020; Lin, 2020). In essence, risks from COVID‐19 seem to have, at least temporarily, changed the relative importance of the information we seek. Changes in density and behavior (Brown et al., 1999; Lima & Dill, 1990) in response to fear of predation have been common in ecological literature. However, demonstrating that individuals of any species fundamentally change how they value different types of information is difficult to measure (Clinchy et al., 2011). Understanding these patterns and processes in humans can inform our understanding of the role emotion can play in natural communities (Clinchy et al., 2011; Frey et al., 2014). These data provide solid evidence that understanding how humans interact with their environment and other organisms may provide us with rare opportunities to identify ecological and evolutionary mechanisms not easily measured in other organisms.

## 4 THE PANDEMIC HAS TRANSFORMED HOW WE APPROACH TEACHING AND LEARNING

Another unique way that our behavior shifted in response to the pandemic was to change how we transmit new information to one another, a phenomenon not well‐articulated in other species. Avoidance of COVID‐19 infection has led to a widespread change in the way we approach teaching and learning. Psychological stressors affecting cognition in animals can be similar in humans (Clinchy et al., 2013). While there is less information on how fear affects how or what animals teach each other, COVID‐19 has certainly changed human behavior in this capacity. The pandemic has caused an abrupt shift in content delivery across disciplines and globally, an alignment in interest among educators that is unprecedented (e.g., Bao, 2020; Basilaia & Kvavadze, 2020; Crawford et al., 2020; Zhang et al., 2020). This alignment may have been predicted from ecological theory as risk (e.g., risk of predation, risk of infection), which tends to constrain decisions to what is safe for the at risk (Hutchinson & Gigerenzer, 2005; Kemp, 2005; Winnie et al., 2006). After all, the fitness consequence of being eaten (or fatally infected in this case) is more severe than missing an opportunity to learn, or procure resources or reproduce for that matter. Because the pandemic forced the abrupt shutdown of universities for safety reasons, in effect it forced all disciplines to move online (Crawford et al., 2020) for “emergency remote instruction” (Hodges et al., 2020). Thus, among educators, COVID‐19 has simplified how we approach learning in a sense by giving us all a common goal of moving to emergency remote instruction.

**Figure 2:**
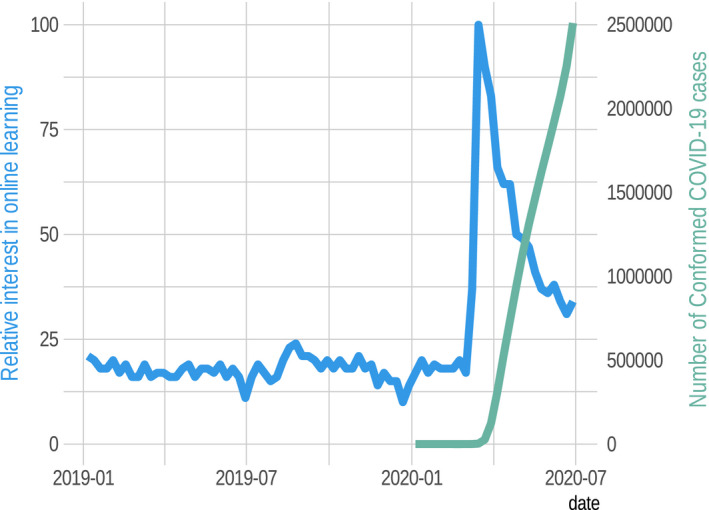
The figure shows how interest in online learning, measured as relative number Google searches for “online learning” beginning January 2019 up to June 30 2020 in the USA, increased exponentially in parallel to the cumulative number of confirmed COVID‐19 infections. Data used in the figure are available at https://github.com/maacevedo/covid19gtrends

Although measures may have been similarly applied across disciplines, the effects of moving online across disciplines are asymmetrical. Disciplines that require human contact such as medicine (Ahmed et al., 2020; Rose, 2020) are perhaps most severely affected. However, field‐based disciplines such as ecology, evolution, and conservation biology are also dramatically affected (Corlett et al., 2020). Although the pandemic has affected us all, different institutionalized and individual strategies to accommodate the threat and burden on education have emerged (e.g., Bao, 2020; Basilaia & Kvavadze, 2020; Crawford et al., 2020; Zhang et al., 2020). The unifying theme is that education has transitioned, rapidly and without time for much preparation, to online delivery, regardless of other nuances in responses or the discipline being affected. Interestingly, like the famous hockey stick graph in climate change literature (i.e., Mann et al., 1999), this unification of interest in online learning is reflected in an abrupt upturn in Google search interest—one which nearly perfectly parallels that of new COVID‐19 infections per week, at least up until mid‐May when classes end for the academic year in many parts of the world (Figure 2). This correlation suggests that interest in strategies for effective remote instruction increased as risk from COVID‐19 increased; again, we used Google search interest and number of new infections as proxies for interest in the topic overall and perceived risk of infection.

Although aspects of our response have been unified, the pandemic has also highlighted inequities in many aspects of our daily experiences (Ahmed et al., 2020), such as access to health care (Hooper et al., 2020), and job and housing security (Raifman & Raifman, 2020; Yancy, 2020). COVID‐19 has also magnified some educational inequities, whereby those previously disadvantaged or at risk of attrition find themselves even more disadvantaged and at greater risk. Students from lower socioeconomic backgrounds are more likely to face challenges from, for example, unreliable internet, added demands of child‐ and elder‐care, job loss, or the need to work as “essential” employees—furthering their risk of contracting COVID‐19 (CDC, 2020; Winter, 2020). In many places where we work, low socioeconomic status overlaps strongly with ethnic and racial categories; consequently, the students we most need to retain are also the most likely to be at greatest risk, including from an educational standpoint with inclusion and retention, during the pandemic. Thus, an added challenge for educators is to not only teach using novel delivery modalities, but also to redouble our efforts at inclusive teaching.

## 5 THE GOALS OF THIS SPECIAL ISSUE

This pandemic has forced many—if not most—of us to reconsider how we communicate information. The Academic Practice section of *Ecology and Evolution* was developed to explore how we do things in our disciplines (Moore et al., 2017). This special issue of Academic Practice provides a forum for our community to present experiences and ideas about the best practices for moving our disciplines online in a focused, accessible, and clearly organized manner. The ultimate purpose was “to provide a rapid outlet to share timely innovations and discoveries for online teaching and learning in ecology and evolution.” However, the pandemic has reached every corner of our society and will change more than just course delivery. Thus, this special issue also covers issues related to meetings and conferences, development of scientific products, strategies to promote open science, how we can best promote equity, diversity and inclusion in the transition to remote instruction, and engaging nonacademic audiences. We wanted the contributions to be a resource for the ecology and evolutionary biology communities as a whole as we adapt to the COVID‐19 crisis, so we emphasized rapid handling of manuscripts, short contributions, and inclusion of tools and resources that facilitate adoption. We have already accepted contributions across a gamut of topics related to how the pandemic has affected learning in our field. Authors contributed on topics related to how the pandemic affected students and instructors (e.g., Barton, 2020) and affected the utility of citizens science (e.g., Smith & Hamed, 2020). Authors contributed thoughtful and creative ways to use the pandemic to stimulate interest in ecology and evolution for students (e.g., Hsu, 2020), providing guidelines, insights, and instructions to safely maintain experiential or active learning or teach topics not well‐suited to online delivery (e.g., Acevedo, 2020; Creech & Shriner, 2020; Hines et al., 2020; Lashley & McCleery, 2020), contributions highlighting sources of inequity and strategies to be more inclusive in online delivery (e.g., Brandt et al., 2020), and ideas for use of tools, apps, and novel media to enhance engagement (e.g., Holt et al., 2020; Strickland et al., 2020). The COVID‐19 crisis demanded action, and with over 40 submissions to this special issue by our community, you have risen to the occasion. We applaud members of our community for contributing their ideas and experiences in this forum and hope that this special issue provides a start to a rich literature base to take learning online in ecology and evolution and beyond.

## CONFLICT OF INTEREST

None declared.

## AUTHOR CONTRIBUTION

**Marcus A Lashley:** Conceptualization (lead); Data curation (supporting); Formal analysis (supporting); Visualization (supporting); Writing‐original draft (lead); Writing‐review & editing (lead). **Miguel Acevedo:** Conceptualization (supporting); Formal analysis (lead); Visualization (lead); Writing‐review & editing (supporting). **Sehoya Cotner:** Conceptualization (supporting); Formal analysis (supporting); Visualization (supporting); Writing‐original draft (supporting); Writing‐review & editing (equal). **Christopher J Lortie:** Conceptualization (supporting); Formal analysis (supporting); Visualization (supporting); Writing‐original draft (supporting); Writing‐review & editing (equal).
